# Selective activators of protein phosphatase 5 target the auto-inhibitory mechanism

**DOI:** 10.1042/BSR20150042

**Published:** 2015-06-22

**Authors:** Veronika Haslbeck, Adrian Drazic, Julia M. Eckl, Ferdinand Alte, Martin Helmuth, Grzegorz Popowicz, Werner Schmidt, Frank Braun, Matthias Weiwad, Gunter Fischer, Gerd Gemmecker, Michael Sattler, Frank Striggow, Michael Groll, Klaus Richter

**Affiliations:** * Department of Chemistry, Center for Integrated Protein Science, Technische Universität München, 85748 Garching, Germany; ‡ Institute of Structural Biology, Helmholtz Zentrum München, 85764 Neuherberg, Germany; † AG Neurodegeneration and Intervention Strategies, German Center for Neurodegenerative Diseases, 39120 Magdeburg, Germany; § Max Planck Research Unit for Enzymology of Protein Folding, 06120 Halle (Saale), Germany

**Keywords:** modulation of phosphatase activity, protein phosphatase 5, small-molecular activators

## Abstract

Protein phosphatase 5 (PP5) is an evolutionary conserved serine/threonine phosphatase. Its dephosphorylation activity modulates a diverse set of cellular factors including protein kinases and the microtubule-associated tau protein involved in neurodegenerative disorders. It is auto-regulated by its heat-shock protein (Hsp90)-interacting tetratricopeptide repeat (TPR) domain and its C-terminal α-helix. In the present study, we report the identification of five specific PP5 activators [PP5 small-molecule activators (P5SAs)] that enhance the phosphatase activity up to 8-fold. The compounds are allosteric modulators accelerating efficiently the turnover rate of PP5, but do barely affect substrate binding or the interaction between PP5 and the chaperone Hsp90. Enzymatic studies imply that the compounds bind to the phosphatase domain of PP5. For the most promising compound crystallographic comparisons of the apo PP5 and the PP5–P5SA-2 complex indicate a relaxation of the auto-inhibited state of PP5. Residual electron density and mutation analyses in PP5 suggest activator binding to a pocket in the phosphatase/TPR domain interface, which may exert regulatory functions. These compounds thus may expose regulatory mechanisms in the PP5 enzyme and serve to develop optimized activators based on these scaffolds.

## Abstract (precis)

This paper describes the identification of compounds, which stimulate the activity of the protein phosphatase PPH-5 and addresses the influence of the identified compounds on the enzymatic properties and the potential mechanism of their action.

## INTRODUCTION

Protein phosphatases, like protein kinases, represent molecular switches within a wide spectrum of signalling cascades. Protein phosphatase 5 (PP5) belongs to the family of phosphoprotein phosphatases (PPPs) with homologous phosphatase domains, like PP1, PP2A, PP2B and others. PP5 is unique among the PPPs in that it harbours a TPR-domain, which serves as docking site for the molecular chaperone heat-shock protein (Hsp90). Early studies have identified PP5 as part of Hsp90–glucocorticoid receptor (GR) complexes [1], where PP5 was shown to dephosphorylate GR and to affect the translocation of the hormone-activated complex into the nucleus [2–4]. PP5 also dephosphorylates the kinases ASK1 (apoptosis signal-regulating kinase 1) and DNA—PKC (protein kinase C) after DNA-damage or during cell-cycle control. In addition, PP5 is associated with the G_1_/S-phase checkpoint regulators ATM and ATR (ataxia telangiectasia and Rad3-related) [5–9]. Hyperphosphorylated tau can also be dephosphorylated by PP5 and other PPP family members [10,11], preventing the misfolded state [12–14] that is relevant for the development of neurofibrillar tangles in tauopathies such as Alzheimer's disease (AD) [15–17]. Interestingly activity levels of PP5 have been found to be reduced in brains of AD patients [10], making higher phosphatase activities desirable. Despite this, attempts to target phosphatase activities have so far mostly focused on the development of inhibitors for PP2A, which plays a role in pancreatic cancers. With LB-100, a potent inhibitor of PP2A recently entered clinical trials [18–22].

The phosphatase activity of PP5 is regulated in a complex activation mechanism by its N-terminal Hsp90-interacting tetratricopeptide repeat (TPR) domain and its C-terminal α-helix, called αJ-helix [23,24]. Moreover, PP5 is regulated by binding to the molecular chaperone Hsp90 [24–26]. In the absence of Hsp90, PP5 exhibits low basal activity. The C-terminal αJ-helix binds to the N-terminal TPR domain of PP5 and locks the enzyme in a latent state [24]. With addition of Hsp90, the C-terminal MEEVD-peptide of the chaperone binds to and stabilizes the phosphatase's TPR domain [1,27,28]. The Hsp90–PP5 interaction disrupts the intramolecular contacts between TPR and αJ-helix and gives access to the active site. Hence, the phosphatase activity of PP5 is stimulated and Hsp90-interacting proteins, like the kinase-dedicated Hsp90-cochaperone Cdc37 (cell-division cycle), can be dephosphorylated with higher rates [29].

Whereas *in vivo* studies imply that enhanced PP5 activities could be beneficial in AD and other human diseases, few synthetic activators of PP5 have been described to date. Only the cell signal transmitter arachidonic acid and certain derivatives thereof are known to stimulate PP5 [25,30,31], but the physiological relevance so far remains elusive. In the present study, we use a synthetic library to identify small-molecule compounds that activate PP5. We then analyse their effect on the enzymatics of PP5 and define the mechanism of PP5 stimulation.

## EXPERIMENTAL

### Materials

The compound library New Chemistry and Discovery Chemistry Collection consisting of 10000 compounds was obtained from ChemDiv. Substances PP5 small-molecule activator (P5SA)-1, P5SA-2, P5SA-3, P5SA-4 and P5SA-5 were purchased from ChemDiv. The C-terminal Hsp90/DAF-21 peptide (AEEDASRMEEVD) was obtained from Biomatik.

### Protein purification

Cloning and purification of PPH-5 (protein phosphatase homologue), CeHsp90/DAF-21 (abnormal DAuer formation), YFP-CeHsp90/DAF-21, BAG-1 (Bcl-2 associated athanogene-1), DNJ-13 (DNJ-13 homologue) and CeHsc70 (*C. elegans* heat shock cognate 70) were performed as described before [32–35]. The human phosphatases PP1, PP2A and PP2B/PP3 were purified as described [36–38]. The expression clones of rat PP5, PP5-ΔN165 (166–499) and PP5-ΔC8 (1–491) were generated by PCR and insertion of the DNA into the pET28 vector. Expression clones of PP5 428–430, which contains A^428^-A^429^-A^430^ instead of E^428^-V^429^-K^430^ and PP5 428–430/458–460, which contains A^428^-A^429^-A^430^ instead of E^428^-V^429^-K^430^ and A^455^-G^456^-A^457^ instead of M^455^-G^456^-N^457^, were also generated by PCR using primer sequences containing the modified codons. PCR-products were inserted into the pET28b expression plasmid (Merck) and verified by DNA sequencing (GATC Biotech). Proteins were expressed in BL21-CodonPlus (DE3)-RIL bacteria (Stratagene). Bacterial cultures were grown to a *D*_600_ of 0.8 and induced with 1 mM IPTG. Cells were disrupted using a cell disruption machine (IUL Instruments) and the lysate was loaded on to a HisTrap FF column (GE Healthcare). Protein was eluted in a buffer containing 400 mM imidazole. Eluted protein was dialysed and applied to a ResourceQ anion exchange column (GE Healthcare). Finally, proteins were subjected to size exclusion chromatography on Superdex 200 PrepGrade gel filtration columns (GE Healthcare). Protein purity was assessed by SDS/PAGE and the molecular mass was determined on an UltraFlex III MALDI-TOF/TOF mass spectrometer (Bruker). Proteins were frozen in liquid nitrogen in 40 mM HEPES/KOH, pH 7.5, 20 mM KCl, 1 mM DTT and stored at −80°C.

### Phosphatase assay during screening and rescreening and characterization

The compound library consisting of 10000 compounds (New Chemistry and Discovery Chemistry Collection, ChemDiv) was screened using a chromometric phosphatase assay with para-nitrophenyl phosphate (pNPP) as substrate. Phosphatase activity was determined by the conversion of pNPP to para-nitrophenole and absorption of the product at 410 nm (ε: 15100 cm^−1^ M^−1^). Assays were performed in 96-well plates in a PerkinElmer EnVision instrument with 50 nM of PPH-5. Reaction volume was 100 μl in a buffer of 40 mM HEPES, pH 7.5, 20 mM KCl, 5 mM MnCl_2_ and 1 mM DTT. The reaction was started by adding pNPP to a final concentration of 60 mM. The slope of the reaction was determined and deviations of >30% were considered potential hits leading to a collection of 76 initial compounds. The Z-factor of the initial screen was calculated from

$$Z=\frac{3\;\times \;{(\sigma }_{P}\;+\;\sigma_{N})}{\left|\sigma_{P}\;-\;\sigma_{N}\right|}$$

where by σ_P_ was 0.3, σ_N_ was 0.1, μ_P_ was 1.6 and μ_N_ was 1 resulting in a Z-factor of −1, indicating that many of the initial compounds will drop out later [39]. These 76 compounds were maintained and rescreened individually in a Varian Cary 100 spectrophotometer (Agilent Technologies) in Hellma SUPRASIL precision quarz cells (10-mm path length) and 140 μl of reaction volumes. Finally, five compounds were obtained as reproducible activators and selected for further characterization.

Phosphatase assays for characterization of PP5 and PPH-5 properties were performed in 40 mM HEPES/KOH, 20 mM KCl, 5 mM MnCl_2_, 1 mM DTT, pH 7.5 and 60 mM pNPP at 20°C if not indicated otherwise. Phosphatase concentrations ranged from 50 to 500 nM in the assay. If indicated, the CeHsp90/DAF-21 peptide AEEDASRMEEVD was supplemented in the assay at a concentration of 60 μM. Compounds were added at concentrations below the occurrence of solubility problems. These concentrations were 70 μM for P5SA-1, 140 μM for P5SA-2, 50 μM for P5SA-3, 100 μM for P5SA-4 and 45 μM for P5SA-5.

### Analytical ultracentrifugation

Binding of Hsp90 to PPH-5 was analysed by analytical ultracentrifugation with fluorescence detection as described before [40]. Experiments were performed in a Beckman ProteomeLab XL-A analytical ultracentrifuge (Beckman) equipped with an AU-FDS detector (Aviv Biomedical). Analytical ultracentrifugation (AUC) runs were performed in 40 mM HEPES/KOH, pH 7.5, 20 mM KCl, 1 mM DTT. The concentration of labelled YFP–Hsp90 was 300 nM and 3 μM of PPH-5 were added to form a complex with YFP–Hsp90. Where indicated 60 μM of the C-terminal Hsp90/DAF-21 peptide (AEEDASRMEEVD) was present in the sample. Centrifugation was performed at 20°C and 142000 ***g*** for 12 h. Scans were recorded every 90 s. Data analysis was performed using a dc/dt approach according to Stafford [41]. The dc/dt plots were fit to Gaussian functions in order to obtain the s_20,w_ values of the respective species as described previously [33]. P5SAs were added as indicated.

### ATPase assay

The ATPase activities of Hsc70 and Hsp90 from *Caenorhabditis elegans* were assessed in a coupled regenerative ATPase assay as described before [32]. Reactions were measured in a Varian Cary 100 spectrophotometer (Agilent Technologies) at 30°C and the depletion of NADH was recorded at 340 nm. Chaperone concentrations were 3 μM and cochaperones were added at a concentration of 5 μM.

### NMR measurements

NMR experiments were performed on an 800 MHz spectrometer (Bruker) with cryoprobe. The measurements were done in ^2^H_2_O with phosphate buffer (4 mM KH_2_PO_4_, 16 mM Na_2_HPO_4_, 120 mM NaCl, pH 7.4) and 1%–5% of d6-DMSO. P5SA-2 without protein was recorded in high sensitivity experiments with a high number of scans. The CPMG sequence with an additional Watergate to suppress residual water was used [42,43]. The T_2_ filtering time was set to 400 ms to suppress signals from slowly tumbling molecules. Peak intensities were normalized by comparing residual DMSO peaks. PPH-5 was added as indicated.

### Crystallization and structure determination

Rat PP5 protein was concentrated to 15 mg/ml in 10 mM Tris, pH 7.8, including 3 mM DTT. Optionally, the ligand was added to a final concentration of 1 mM. Crystals were grown at 20°C within 4 weeks by using the hanging drop vapour diffusion method. Drops contained equal volumes of protein and reservoir solutions [0.2 M Mg(NO_3_)_2_, 20% PEG 3350]. Crystals were soaked for 30 s in cryo buffer (mother liquor+25% PEG 200) and were subsequently cooled in liquid nitrogen at 100 K. Diffraction datasets were recorded using synchrotron radiation of λ=1.0 Å (1 Å=0.1 nm) at the beamline X06SA, Swiss Light Source (SLS). Collected datasets were processed using the program package XDS [44].

Determination of the crystal structure was performed by molecular replacement using the program PHASER [45]. Human PP5 (PDB ID: 1WAO) [24] was applied as starting model for the ligand structure PP5–P5SA-2. The refined coordinates of PP5–P5SA-2 in turn were employed for the apo-structure of PP5. Model building was carried out with the graphic program MAIN [46] and finalized applying REFMAC5 [47] by conventional crystallographic rigid body, positional and anisotropic temperature factor refinements with current crystallographic values of *R*_work_=23.8%, *R*_free_=27.1%, RMSD bond lengths of 0.005 Å and RMSD bond angle of 0.99° for PP5 apo and *R*_work_=21.4%, *R*_free_=26.1%, RMSD bond length of 0.015 Å and RMSD bond angle of 1.68° for PP5–P5SA-2.

Coordinates were confirmed to have good stereochemistry indicated by the Ramachandran plot. The atomic co-ordinates have been deposited at the RCSB Protein Data Bank under the accession codes 4JA9 for PP5 apo and 4JA7 for PP5–P5SA-2. The amino acid numbers in the text reflect the full-length PP5 protein of *Rattus norvegicus*.

## RESULTS

### Identification of novel small molecule activators of PPH-5

**Figure 1:**
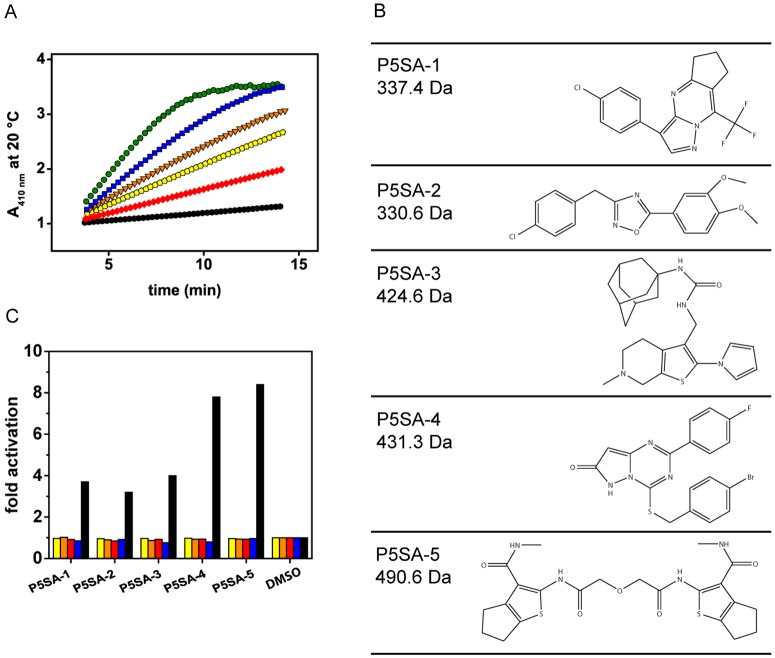
Small molecules selectively activate nematode PPH-5 (**A**) Phosphatase assays were performed with PPH-5 and five identified compounds. The substances (50 ng/ml) P5SA-1 (yellow), P5SA-2, (orange), P5SA-3, (red), P5SA-4 (blue) and P5SA-5 (green) activate PPH-5 (DMSO control in black). (**B**) Chemical structures of the five activators. (**C**) Phosphatase assays of human PP1 (yellow), PP2A (orange) and PP2B/PP3 (red), as well as the BIAP (blue). For comparison, PPH-5 is depicted in black. Compound concentrations were the maximal stable concentrations outlined in the materials and methods section. Results are expressed as mean ± S.D. (*n*≥3).

PP5 is a highly regulated enzyme. The auto-inhibition of the phosphatase is controlled by the N-terminal TPR domain and the C-terminal αJ-helix, implying a complex mechanism to control the turnover rate [24]. We aimed at identifying small molecules which can alter the activity of the phosphatase as these compounds may target the specific rate-controlling mechanism of PP5. PP5 from nematode origin (PPH-5), which contains all the regulatory features of the human protein (Supplementary Figure S1), was used to screen a library of 10000 compounds in 96-well plates. Using the substrate pNPP, we analysed the activity of PPH-5 in the presence of compounds and initially retained 76 molecules from the primary screen. Each of the identified candidates was individually tested resulting in five compounds which reproducibly increase the enzymatic activity of PPH-5 (Figure 1A). All five molecules are smaller than 500 Da (Figure 1B), are stable at room temperature and follow the Lipinsky's rules making them good candidates for further optimization. These P5SAs (P5SA-1–5) were applied at different concentrations to determine the apparent *K*_D_-value in the pNPP-based assay yielding constants in the range of 6 to 26 μM and a 3–8-fold activation compared with the basal activity (Table 1; Supplementary Figure S2). In particular, P5SA-2 and P5SA-5, with apparent affinity constants of 7.8 and 6.4 μM, displayed a reasonable high affinity and sufficient solubility enabling an enzymatic analysis of their activation mechanisms.

**Table 1:** The enzymatic parameters of nematode PPH-5 and mammalian PP5 are influenced by the P5SAs

	PPH-5 (*C. elegans*)				PP5 (*R. norvegicus*)	
	*K*_D_ (μM)	*K*_M_ (mM)	*k*_cat_ (s^−1^)	fold activation	fold activation	
DMSO	–	7.0±0.4	0.62±0.17	–	–	–
P5SA-1	13.3±4.2	3.0±0.2	2.28±0.28	3.7	4.1	at 100 μM
P5SA-2	7.8±1.4	2.5±0.1	1.99±0.43	3.2	3.9	at 100 μM
P5SA-3	n.d.	n.d.	n.d.	4.0	2.9	at 30 μM*
P5SA-4	25.7±5.5	5.0±0.3	4.85±0.43	7.8	3.0	at 100 μM
P5SA-5	6.4±1.8	4.3±0.3	5.22±0.67	8.4	8.6	at 100 μM

Enzymatic parameters were determined using the substrate pNPP in phosphatase assays. Reaction buffer was 40 mM HEPES/KOH, pH 7.5, 20 mM KCl, 5 mM MnCl_2_ and 1 mM DTT. pNPP concentrations were 60 mM with exception of the *K*_M_-determination, where pNPP-concentrations were varied. The ‘fold activation’ is calculated based on the *k*_cat_ values compared with PPH-5. Used compound concentration are indicated (*P5SA-3 solubility was not sufficient to titrate fully and precipitation occurred at 30 μM). n.d., not determined

### Small-molecule activation is highly specific to PP5-like proteins

We first addressed the specificity of the identified activators by analysing unspecific effects on the ATP hydrolysing activity of the ATPases Hsc70 and Hsp90. The P5SAs neither affected the activity of the chaperones, nor abrogated the binding of cofactors to Hsc70 or Hsp90 (Supplementary Figure S3), showing that the compounds do not generally affect protein activities. Next we tested, whether the compounds affect other phosphatases. Since other PPP family members contain phosphatase domains with high sequence similarity, we examined PP1, PP2A and PP2B/PP3. Neither activation nor inhibition of the respective phosphatase's activity was detected, implying that the identified molecules are specific activators of PPH-5 (Figure 1C). Also bovine intestinal alkaline phosphatase (BIAP) was not affected by the compounds. We then challenged homologues of PPH-5 from other species. Even at high compound concentrations we could not detect activation of the yeast homologue Ppt1 which harbours an identity of 31% with PPH-5 (Supplementary Figure S1). However, PP5 from *R. norvegicus* with 67% sequence identity to nematode PPH-5 and 98% identity to the human protein exhibited activation when treated with either of the P5SAs at a high compound concentration (Table 1).

### The P5SAs are allosteric regulators of PPH-5

To gain insight into the activation mechanism of P5SAs, we investigated the interaction between the substrate pNPP and the P5SAs during the dephosphorylation reaction. Direct correlation between the small molecules during the hydrolysis of pNPP should be reflected in a lower apparent *K*_M_-value for pNPP in the presence of the activators. Instead, all P5SAs strongly increase the *k*_cat_ value, but only weakly influence the *K*_M_-value for pNPP (Supplementary Figure S4; Table 1). Hence, there is only limited influence of the compounds on the substrate pNPP; instead, all compounds act as allosteric activators of PPH-5.

To test whether the activators target different steps during the enzymatic reaction, we wondered whether they stimulate in an additive manner. To address this, we measured the combinatorial effects of the P5SAs on the stimulation of pNPP hydrolysis. Compound concentrations of three times of the apparent *K*_D_ value were used by combining the different activators. The observed activities revealed no additional stimulation and complied with the estimations of individual but non-cooperative interaction (Supplementary Figure S5). These results suggest that all identified P5SAs accelerate the rate-limiting step during the enzymatic cycle, even though they may use very different binding strategies to achieve this given the differences in their structure.

**Figure 2:**
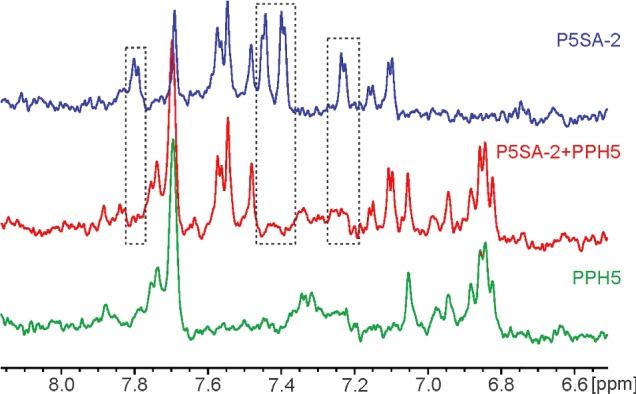
NMR-analysis of PPH-5 interaction with P5SA-2 NMR analysis of P5SA-2 confirms binding of P5SA-2 to PPH-5. P5SA-2 specific peaks (blue) disappear upon addition of 10 μM of PPH-5 (red). The PPH-5 spectrum is shown below for comparison (green). Missing peaks indicate decrease in the P5SA-2 tumbling rate caused by binding to PPH-5. Peaks that do not disappear after protein addition indicate that certain moieties of P5SA-2 are not directly immobilized by PPH-5. Peaks ~7.7 ppm originates from buffer impurities. Measurements were performed in phosphate buffer at 20°C as outlined in the ‘Experimental’ section.

Allosteric modulators should be able to bind their target protein in the absence of substrate. To confirm direct binding to PPH-5, we employed NMR spectroscopy. We recorded relaxation-edited NMR spectra of P5SA-2 (Figure 2, upper panel). We then added PPH-5 at a concentration of 10 μM. Relaxation-editing strongly attenuates signals from big, slowly tumbling molecules but retains signals from small, fast tumbling moieties [42]. Most of the peaks of P5SA-2 disappeared after addition of PPH-5 (Figure 2). This reflects a much slower tumbling rate of the protein–ligand complex in comparison with the free molecule. Some compound peaks remained unaffected though, indicating that certain moieties of the P5SA-2 molecule are still able to rotate freely even in complex with PPH-5.

### Hsp90 binding is not compromised by the small molecules

**Figure 3:**
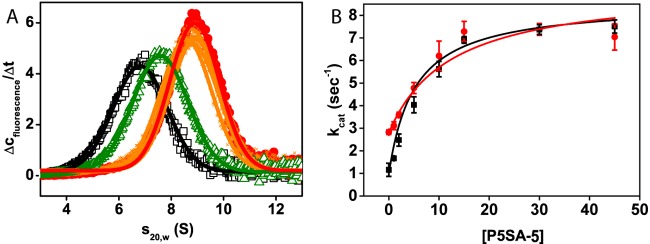
PP5–Hsp90 complex formation is unaffected by the P5SAs (**A**) Complex formation between PPH-5 and CeHsp90/DAF-21 is not influenced by the presence of the P5SAs. The sedimentation coefficient s_20,w_ of YFP–Hsp90 is shifted from 6.8 S (black) to 8.9 S in presence of PPH-5 (red). Supplementation of different P5SAs (orange) does not alter the sedimentation coefficient of the phosphatase–chaperone complex. Presence of the Hsp90-derived MEEVD peptide shifts the s_20,w_ value from 8.9 to 7.6 S (green). P5SAs were added at their maximal soluble concentrations (**B**) Titration of PPH-5 phosphatase activity with P5SA-5 in absence of MEEVD peptide is depicted in black whereas activities in presence of 60 μM peptide are shown in red. Results are expressed as mean ± S.D. (*n*≥3).

To understand the stimulation mechanism, we then investigated whether stimulation by P5SAs and stimulation by Hsp90 interfere. To this end, we analysed, whether the P5SAs reduce the binding of Hsp90 to the TPR domain. Binding of PPH-5 to YFP-tagged Hsp90 was addressed by analytical ultracentrifugation in sedimentation velocity experiments. In these experiments, YFP–Hsp90 shows a sedimentation coefficient of 6.8 S. Upon complex formation with PPH-5 the sedimentation coefficient of YFP–Hsp90 is increased to 8.9 S enabling analysis of the binding reaction between these two proteins (Figure 3A). This binding event was also observed in the presence of the maximal soluble concentration of each P5SA, where none of the P5SAs visibly reduced the complex formation. We then used the C-terminal MEEVD-containing peptide of Hsp90 as control to disrupt the complex between PPH-5 and YFP–Hsp90. Indeed, complex formation is strongly reduced under these conditions.

To confirm this from a different perspective, we were interested whether the compound-induced stimulation is affected in the presence of the TPR-binding peptide. Thus we tested the stimulatory potential of the activator P5SA-5 in the presence of the MEEVD-containing peptide. P5SA-5 was chosen, as it shows the highest affinity and stimulation factors among the activators making these measurements possible. With 60 μM of the peptide supplemented, a large excess of MEEVD-containing peptide was used. In the phosphatase assay only a minor influence on the apparent affinity of P5SA-5 was observed despite the high concentration of MEEVD-containing peptide (Figure 3B). Thus, this P5SA appears to utilize a site distinct from the peptide-binding groove of the TPR domain to exert its stimulatory potential.

### The P5SAs bind to the isolated phosphatase domain of PPH-5

**Figure 4:**
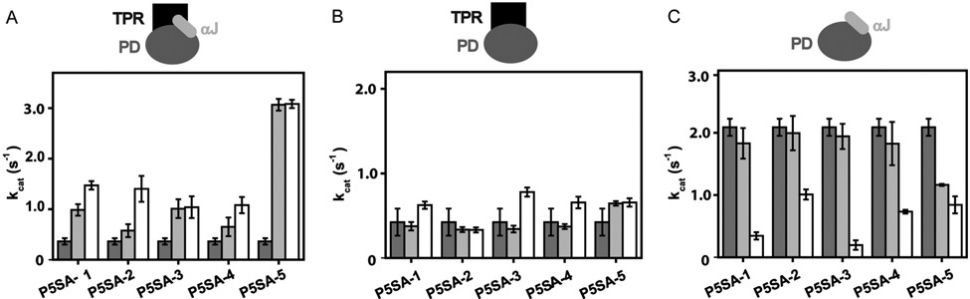
P5SAs interfere with the auto-regulatory mechanisms of mammalian PP5 Phosphatase assays were performed with rat PP5 and variants thereof (**A–C**). Conditions were as outlined in the ‘Experimental’ section. Activity of PP5 in the absence of compound (dark grey) or in the presence of 10 μM (light grey) or in the presence of 100 μM (white bar) of the respective P5SA. (**A**) Activation of full-length PP5, (**B**) PP5-ΔC8 and (**C**) PP5-ΔN165 in the presence of P5SAs. Results are expressed as mean ± S.D. (*n*≥3).

To determine, which parts of the protein are required to obtain the stimulation from the five different P5SAs, we utilized deletion constructs of PP5. The deletion constructs of rat PP5 were generated based on previously published constructs of the highly homologous human protein [24]. The stimulatory potential was recorded by applying the activators at 10 μM and 100 μM (or maximal soluble concentration). Only in the case of P5SA-5, an effect at the lower concentration was detectable, suggesting that the binding preference of the other compounds towards the mammalian PP5 is rather low (Figure 4A; Table 1).

As first mutant, we generated a PP5 protein which lacks the last eight amino acids including Gln^495^ (PP5-ΔC8) which is supposedly involved in maintaining the auto-inhibition of PP5 [30]. The turnover of PP5-ΔC8 with 0.42 s^−1^ is similar to full-length rat PP5. Whereas the deletion of this helix apparently barely influences the enzymatic turnover, the effect on the potential of the compounds was striking. We did not observe a stimulation of the PP5-ΔC8 mutant, as addition of either P5SA did not activate PP5-ΔC8 (Figure 4B) implying that some kind of regulatory function had been altered.

Next we generated a mutant, which lacks the entire auto-inhibitory TPR-domain (PP5-ΔN165). The deletion of the TPR-domain in PP5-ΔN165 indeed diminished the auto-inhibition. Compared with the full-length protein, the isolated phosphatase domain showed an increased activity (2.09 s^−1^ compared with 0.36 s^−1^). Remarkably, whereas wild-type (wt)-PP5 is activated by the P5SAs, the P5SAs inhibit the phosphatase activity of the isolated phosphatase domain by up to 70% (Figure 4C). Given that the TPR-domain is not part of this construct, this effect illustrates that the P5SAs can interact with the phosphatase domain of PP5. This distinguishes our activators from established ones, which target the TPR domain [31]. The inhibitory effect towards the phosphatase domain further demonstrates that the allosteric regulation sites can influence the activity of the hydrolysing protein in both directions. In the presence of the full auto-inhibitory domain, P5SAs stimulate the phosphatase activity, in the absence of the self-inhibitory effect of the TPR-domain, their binding to the highly-activated phosphatase domain reduces the turnover rate.

### P5SA-2 causes a tilting at the TPR-phosphatase interface

To analyse the mode of interaction directly we solved at molecular resolution the structures of rat PP5 in its apo-state as well as in presence of the ligand P5SA-2. Starting phases were obtained by molecular replacement methods [45] using the coordinates of human PP5 (PDB ID: 1WAO) [24]. The PP5–P5SA-2 dataset were refined to 2.0 Å (*R*_free_=26.1%; PDB ID: 4JA7) whereas the apo-structure yielded a final resolution of 2.3 Å (*R*_free_=27.1%; PDB ID: 4JA9, Table 2). In both structures, the first defined amino acid is Gly^23^. The loop connecting the TPR domain with the phosphatase domain (Arg^150^–Arg^158^) is flexible and therefore was not defined in the electron density map.

**Table 2:** Data collection and refinement statistics

	PP5 apo	PP5 + P5SA-2
Crystal parameters		
Space group	P4_1_2_1_2	P4_1_2_1_2
Cell constants	a=b=50.1 Å, c=379.2 Å	a=b=51.1 Å, c=365.7 Å
Molecules per AU*	1	1
Data collection		
Beamline	SLS, PXI–X06SA	SLS, PXI–X06SA
Wavelength (Å)	1.0	1.0
Resolution range (Å)^†^	40–2.3 (2.4–2.3)	30–2.0 (2.1–2.0)
Number of observed reflections	140950	286970
Number of unique reflections^‡^	22932	34075
Completeness (%)^†^	99.5 (99.9)	98.5 (93.1)
*R*_merge_ (%)^†^^§^	4.1 (53.7)	3.8 (43.5)
I/σ (I)^†^	22.3 (4.4)	28.8 (5.6)
Refinement (REFMAC5)		
Resolution range (Å)	15–2.3	15–2.0
Number of atoms		
Protein	3757	3749
Water	63	106
*R*_work_/*R*_free_ (%)^║^	23.8/27.1	21.4/26.1
RMSDs^¶^		
Bond lengths/angles (Å)/(°)	0.005/0.99	0.015/1.68
Average B-factor (Å^2^)	74.1	51.0
Ramachandran plot (%)**	95.2/4.6/0.2	95.7/4.3/0
PDB accession code	4JA9	4JA7

*Asymmetric unit.

†The values in parentheses of resolution range, completeness, *R*_merge_ and I/σ (I) correspond to the last resolution shell.

‡Friedel pairs were treated as identical reflections.

§*R*_merge_ (I)=Σ_hkl_Σ_j_ |I(hkl)j–<I_hkl_>|/Σ_hkl_Σ_j_I(hkl)_j_ where I(hkl)_j_ is the j^th^ measurement of the intensity of reflection hkl and <I(hkl)> is the average intensity.

^║^ *R*=Σ_hkl_ | |F_obs_| − |F_calc_| |/Σ_hkl_ |F_obs_|, where *R*_free_ is calculated without a sigma cut-off for a randomly chosen 5% of reflections, which were not used for structure refinement and *R*_work_ is calculated for the remaining reflections.

¶Deviations from ideal bond lengths/angles.

**Number of residues in favoured region/allowed region/outlier region.

**Figure 5:**
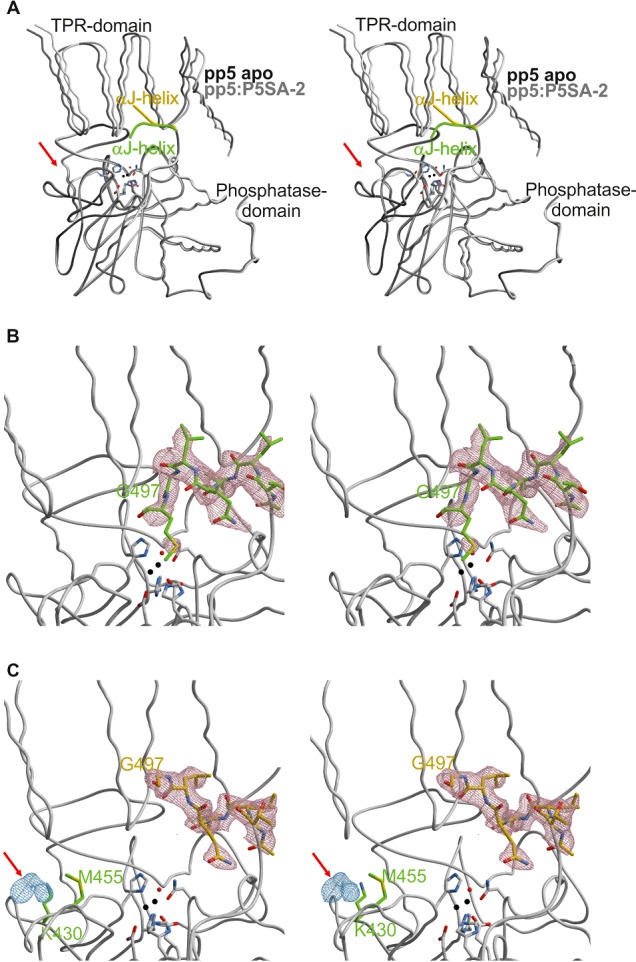
P5SA-2 leads to disintegration of regulatory domain contacts (**A**) Stereo view of the backbone superposition of both molecules shown as ribbon plots: PP5 apo with αJ-helix in dark grey and green, PP5–P5SA-2 with αJ-helix in light grey and yellow. The position of the additional electron density which is only visible in the PP5–P5SA-2 structure is indicated by a red arrow. Active site residues are depicted as sticks and the two magnesium ions (black) and one water molecule (red) are shown as balls [also in (**B**) and (**C**)]. (**B**) Stereo view of the 2F_o_-F_c_ electron density map (countered at 1σ) for the αJ-helix (T^492^–M^498^) of PP5 apo, in which the αJ-helix has been removed for phase calculations. The position of Gly^497^ is indicated. (**C**) Stereo view of the 2F_o_-F_c_ electron density map (at 1σ) for the αJ-helix (T^492^–G^497^) of PP5 in complex with P5SA-2. The electron density depicted in blue at 1σ indicates the potential binding site of the ligand P5SA-2. The indicated regulatory binding pocket for P5SA-2 was confirmed by mutational studies.

A comparison of the backbone traces of the apo and the PP5–P5SA-2 complex discloses limited structural rearrangements within the phosphatase domain, reflected by an RMSD of the Cα atoms of 0.42 Å (Figure 5A). By contrast, a tilting of the TPR domain up to 10° is observed in the ligand complexed structure (RMSD: 0.84 Å) accompanied by a rearrangement of the αJ helix. These structural domain reorganizations are manifested in a reduction in the crystal lattice constant c by 15 Å. Inspecting the omit maps for both αJ helices ranging from Asn^491^ to Gly^497^ or to Met^498^ respectively, a considerable displacement of the Cα-atom of Gly^497^ by 6.5 Å is observed (Figures 5B and 5C). Comparing the structures of the human and rat apo PP5 proteins, we observe a similar positioning of the αJ-helix in both, whereas the TPR domain of the human PP5 is even further tilted relative to the rat PP5–P5SA-2 structure (Supplementary Figure S6). This tilting of the domains and the changes in the αJ helix could well be the reason for the higher activity in the presence of P5SA-2.

The ligand P5SA-2 itself cannot be modelled in the F_0_-F_c_ electron density map. However comparison of the two structures, PP5 apo and the PP5–P5SA-2 complex, assigns a potential binding site of the ligand at the interface between the TPR and the phosphatase domain. The visible parts of the ligand structure imply interactions with the phosphatase domain at amino acids Glu^428^, Val^429^, Lys^430^, Ala^431^, Glu^435^, Met^455^ and Asn^457^. The modulate character of the P5SA-2 interaction would be in agreement with the NMR data of this complex (Figure 2), which suggested that parts of the ligand are not complexed by the protein. In summary, the observed structural rearrangements induced by ligand binding support the functional data on the P5SAs and provide a potential binding site at the phosphatase-TPR domain interface.

### Mutation of the regulatory pocket affects the turnover rate and the activation potential

**Figure 6:**
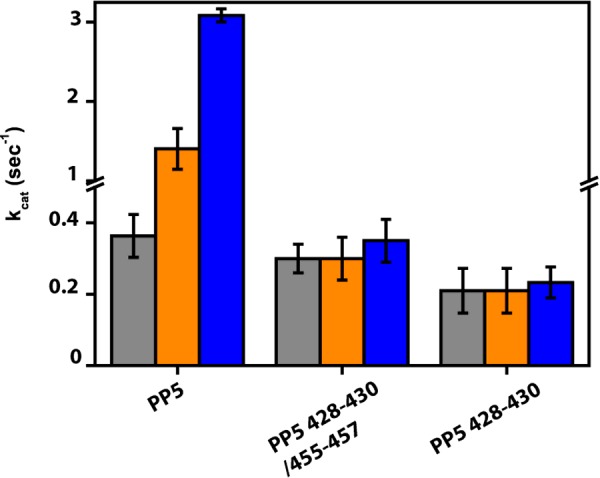
Mutations in the phosphatase domain of PP5 abrogate activation by P5SAs Phosphatase activities of PP5, PP5 428–430/455–457 and PP5 428–430 and are depicted in the absence (grey) and presence of 100 μM P5SA-2 (orange) and 100 μM P5SA-5 (blue) respectively. Results are expressed as mean ± S.D. (*n*≥3).

The crystal structure of PP5 in complex with the activator P5SA-2 exposed the amino acid positions involved in ligand binding. To alter the proposed pocket, we designed two different PP5-variants, in which we changed the amino acids complexed by P5SA-2 to alanine. In the first mutant, we changed the residues of both loops forming the potential binding site. Thus the loop E^428^-V^429^-K^430^ was changed to A^428^-A^429^-A^430^ and the loop M^455^-G^456^-N^457^ was changed to A^455^-G^456^-A^457^, resulting in the protein PP5 428–430/455–457. This protein was slightly destabilized compared with the wt protein (result not shown) and the phosphatase activity was reduced to 0.20 s^−1^. Importantly, we could not observe any stimulation with the activator P5SA-2 (Figure 6) and likewise we could not detect stimulation with P5SA-5.

We then generated a mutant, in which only the loop M^455^-G^456^-N^457^ was replaced. This mutant was as stable as wt protein (result not shown) and its activity was barely affected with a turnover of 0.35 s^−1^. Testing the potential of P5SA-2, no activation could be achieved even at a concentration of 100 μM (Figure 6), implying that indeed this site is important to promote the effect of the activatory compound. We also tested the activator P5SA-5 (Figure 6), which displays the highest affinity to rat PP5 and nematode PPH-5, but was not amendable for X-ray structural analysis. This compound likewise did not stimulate the catalytic activity of either PP5 variant, suggesting that these two compounds target the PP5 enzyme in a similar step.

## DISCUSSION

Targeting phosphatases as potential points of interference in cancer and other diseases has been described and compounds had been identified in the past. The farthest developed compounds, like LB-100, target PP2A for its involvement in various forms of cancer [19–22]. PP5 is unique in its regulation among the protein phosphatases with its complex regulation, which ensures that substrates are dephosphorylated when recruited to the Hsp90 chaperone network [26,29]. It is controlled by an auto-inhibitory mechanism involving its Hsp90-interacting TPR domain and its C-terminal αJ helix [1,28]. In the present study, we report five compounds (P5SAs) that modulate the activity of PP5. Notably, the P5SAs characterized in our study demonstrate a TPR-independent regulation of PP5 activity. This mechanism is distinct from other PP5 activators described so far, including arachidonic acid and its derivatives, which bind to the TPR domain [25,30,31]. The newly found P5SAs do not share much structural similarity, but each P5SA is able to increase significantly the turnover rate of the enzyme. This approach could in principle be helpful in cases, where PP5-activity is reduced or where increased dephosphorylation of its substrate proteins is desirable.

Based on PP5 deletion constructs and X-ray structures we were able to allocate a potential binding region for one compound (P5SA-2) to the phosphatase domain at the interface with the TPR domain. At this junction, residual electron density was observed in the PP5–P5SA-2 complex structure. The F_o_-F_c_ electron density was not sufficient to unambiguously identify the ligand, but this electron density was absent from the apo structure. Substitution of the involved amino acids M^455^-G^456^-N^457^ to A^455^-G^456^-A^457^ and E^428^-V^429^-K^430^ to A^428^-A^429^-A^430^ resulted in loss of the stimulatory potential of the compound P5SA-2, implying that this pocket is indeed involved in regulatory processes, which are altered by the compound. The uncovered binding pocket may thus prove to be one possible checkpoint in the protein structure, where a strong modulatory effect can be obtained. The identified candidates derived here and the regulatory site which is targeted by P5SA-2 is suitable for further optimization and is probably interesting to address various diseases PP5 is involved in.

In respect to the stimulation mechanism, the most significant change upon ligand binding exposes a structural reorganization of the phosphatase's C-terminus and a tilting of single helices of the TPR domain up to 10°. Limited flexibility in the orientation of the two domains had been observed in the human protein before [24]. In our case, these conformational rearrangements apparently are caused by the compound. Our biochemical data imply that the stimulation involves a release of the auto-inhibition of the phosphatase. The flexibility in domain arrangement of the phosphatase may hint to a more complex mechanism of this protein and potential conformational changes upon binding its protein substrates. Based on these results and previous reports, the targeting of PP5 represents a promising new strategy to influence various diseases originating from hyperphosphorylation. One prominent example is AD, where PP5 is involved in the dephosphorylation of tau protein. Other potential applications for PP5 activators include different types of cancer, where dephosphorylation of certain transcription factors or protein kinases is dysfunctional and contributes to the development of the disease. The described compounds may represent a first step in defining the options and possibilities of the approach to target PP5 in order to alter cellular pathways in diseases.

## Abbreviations

AD: Alzheimer’s disease
BIAP: bovine intestinal alkaline phosphatase
GR: glucocorticoid receptor
Hsp: heat-shock protein
P5SA: protein phosphatase 5 small-molecule activator
pNPP: para-nitrophenyl phosphate
PP5: protein phosphatase 5
PPP: phosphoprotein phosphatase
SLS: Swiss Light Source
TPR: tetratricopeptide repeat
wt: wild-type
